# Moving to the Field: Plant Innate Immunity in Crop Protection

**DOI:** 10.3390/ijms18030640

**Published:** 2017-03-15

**Authors:** Marcello Iriti, Elena M. Varoni

**Affiliations:** 1 Department of Agricultural and Environmental Sciences, Milan State University, via G. Celoria 2, 20133 Milan, Italy; 2 Department of Biomedical, Surgical and Dental Sciences, Milan State University, via Beldiletto 1/3, 20142 Milan, Italy; elena.varoni@unimi.it

In natural ecosystems, disease is not the rule, but a rare outcome in the spectrum of plant–microbe interaction, since plants have developed, during their evolutionary history, various defence strategies to face pathogens. Therefore, in this evolutionary arms race, plants have (co)evolved a complex set of defence mechanisms to counteract pathogen challenging and, in most cases, prevent infection. As animals, plants are able to recognize and distinguish between self, non-self, and altered self, by their innate immune system, thus activating a battery of defence reactions. When a pathogen becomes able to overcome these defences, disease ceases to be the exception [1]. Though a comprehensive discussion on the plant immune system is beyond the scope of this editorial, the molecular mechanisms involved in the plant immunity have been recently reviewed [2,3,4,5,6]. Kørner et al. [7] emphasized the cross-talk between endoplasmic reticulum (ER) stress signaling pathways and immune responses in plants. In particular, IRE1 (inositol requiring enzyme 1) is a conserved ER stress sensor protein identified as a transcriptional regulator of ER genes and involved in immunity and programmed cell death (PCD). In their review article, Wang et al. [8] focused on the role of NADPH oxidases, the major source of apoplastic reactive oxygen species (ROS) under both normal and stress conditions, in mediating PCD and plant immune response.

However, with the advent of the agro-ecosystem, this equilibrium was altered because of human activities such as intensive farming, monoculture, and varietal selection. In this context, diseases that damage crops have to be managed by different control strategies integrated into pest management programs.

According to Regulation (EC) N° 1107/2009, a plant protection product generally contains more than one component, and the active constituent against pathogens/pests/weeds is referred to as active substance [9]. Plant protection products are usually used for (i) protecting plants or plant products against damaging organisms; (ii) influencing the plant growth (plant growth regulators); and (iii) preventing growth or eradicating undesired plants (weeds). Nowadays, chemical control represents the most used and effective strategy in crop protection, with a variety of agrochemicals available to control plant diseases, pests, and weeds, such as fungicides, insecticides, and herbicides. In this scenario, the use of elicitors and plant activators represents a novel and promising strategy in crop protection, as an alternative to conventional agrochemicals that exert direct toxic effects on noxious organisms. Indeed, elicitors and plant activators trigger the plant’s own defence mechanisms by stimulating the plant innate immune system, differently from conventional pesticides. Alexandersson et al. [10] provided a current summary of plant resistance inducers that have been successfully used in Solanaceae species to protect against pathogens.

In the EU Pesticide database (http://ec.europa.eu/food/plant/pesticides/eu-pesticides-database) [11], a database on registered active substances in Europe, selecting category-approved fungicides, herbicides, and insecticides, in total 155, 127, and 105 entries can be found, respectively. However, when selecting category-approved elicitors and plant activators, only 7 and 2 entries are available, respectively (Table 1).

Among elicitors, chitosan has been studied so far for its antiviral and antifungal activities. It is a linear, polycationic heteropolysaccharide consisting of two monosaccharide units, *N*-acetyl-d-glucosamine, the repeat unit of chitin, and d-glucosamine. Therefore, chitosan is produced by the deacetylation of chitin, the structural component of fungal cell walls as well as insect exoskeletons. Chitosan treatment mimics a plant–pathogen interaction when, upon host penetration, fungus deacetylates its own cell wall chitin into chitosan to escape plant chitinases. In these terms, chitosan represents a pathogen- or microbe-associated molecular pattern (PAMP or MAMP), i.e., a general (race-nonspecific) elicitor able to prime a nonspecific, long-lasting, and systemic immunity (also known as systemic acquired resistance, SAR) possibly by binding to a putative pattern recognition receptor (PRR) in the plant cell [1]. Luti et al. [12] investigated the PAMP activity of cerato-platanin, a Cys-rich protein produced by the pathogenic ascomycete *Ceratocystis platani*, in Arabidopsis, by an elegant proteomic and volatilomic approach. Among plant activators, acibenzolar-*S*-methyl or benzothiadiazole (*S*-methyl benzo[1,2,3]thiadiazole-7-carbothioate) deserves particular attention. The latter is a functional analogue of salicylic acid, a plant hormone that plays a central role in innate immunity as a co-activator of immunity-induced transcription reprogramming [13].

COS-OGA is an oligosaccharidic complex comprising chitooligosaccharides (COSs) and pectin-derived oligogalacturonides (OGAs). Therefore, this elicitor results from the association of both plant non-self (chitosan, a PAMP, with a mean polymerization degree of 7) and altered self molecules (oligopectates with a mean polymerization degree of 11). In plant immunity, OGAs are damage-associated molecular patterns (DAMPs), i.e., general (race-nonspecific) elicitors that mimic degradation of plant cell wall and middle lamella pectin by fungal polygalacturonases and further fragmentation by plant enzymes [14]. Surprisingly, in EU, COS-OGA is registered as a low-risk fungicide for which no maximum residue levels (MRLs) are required.

At the end of this brief editorial, it appears evident that research activity and studies focusing on plant immunity greatly stimulated the development and registration of plant protection products based on a non-biocide mechanism of action, namely elicitors and plant activators. In general, these formulates are less toxic and more environmentally friendly than conventional agrochemicals, thus meeting the needs of a modern and sustainable agriculture. Noteworthy, these products represent one of the few strategies to control viral diseases [1] and can confer tolerance to abiotic stresses, such as drought, thus contributing to the management of water resources in a global climate change scenario [15]. Not least, priming the plant immune system can serve as a means to increase the content of bioactive phytochemicals in plant foods. In fact, elicitors and plant activators stimulate the plant secondary metabolism and the accumulation of defence metabolites (phytoalexins) in plant tissues, such as polyphenols now recognized as health-promoting components of plant foods [16,17,18]. However, some limitations exist. Elicitors and plant activators can incur fitness costs in crops due to the trade-off between resources allocated for growth and reproduction and for disease resistance, though this strictly depends on the concentrations used and other environmental factors [19,20]. In conclusion, more mechanistic studies are urgently needed to improve basic knowledge on plant immunity, in the hope that this can further inspire the development of new safe plant protection products.

## Figures and Tables

**Table 1:** Elicitors and plant activators approved in the European Union *.

Active Substance (ID) ^^^	Date of Approval	Classification GHS ^‡^	MRLs **	Toxicological Information		
				ADI ^#^ (mg/kg bw/d) ^§^	ArfD ^#^ (mg/kg bw)	AOE ^#^ (mg/kg bw/d)
**Elicitors**						
Chitosan hydrochloride (1096)	01/07/2014	No classification	No MRL required	NA ^†^	NA	NA
Fructose (2375)	01/10/2015	No classification	No MRL required	NA	NA	NA
Heptamaloxylglucan (1449)	01/06/2010	No classification	No MRL required	NA	NA	NA
Laminarin (1510)	01/04/2005	No classification	No MRL required	NA	NA	NA
Pepino Mosaic Virus strain CH2 isolate 1906 (2315)	07/08/2015	No classification	No MRL required	NA	NA	NA
Sucrose (2340)	01/01/2015	No classification	No MRL required	NA	NA	NA
Zucchini Yellow Mosaic Virus weak strain (2020)	01/06/2013	No classification	No MRL required	NA	NA	NA
**Plant activators**						
Acibenzolar-*S*-methyl (benzothiadiazole) (914)	01/04/2016	Skin corrosion/irritation Category 2 (H315) Skin sensitisation Category 1 (H317) Serious eye damage/irritation Category 2 (H319) Specific target organ toxicity single exposure Category 3 (H335) Hazardous to aquatic environment short term/acute Category 1 (H400) Hazardous to aquatic environment long term/chronic Category (H410)	MRLs required ^¥^	0.03	0.03	0.03
Cerevisane (2301)	23/04/2015	No classification	No MRL required	NA	NA	NA

* Source: EU Pesticide database (http://ec.europa.eu/food/plant/pesticides/eu-pesticides-database) retrieved on January 20th 2017; ^^^ Identification number; ^‡^ Globally Harmonized System of Classification and Labelling of Chemicals; ** Minimum Residue Levels; ^#^ ADI: acceptable daily intake; ARfD: acute reference dose; AOEL: acceptable operator exposure level; ^§^ bw: body weight; d: day; ^†^ NA: not applicable; ^¥^ Sum of acibenzolar-*S*-methyl and acibenzolar acid (free and conjugated).
